# VariantClassifier: A hierarchical variant classifier for annotated genomes

**DOI:** 10.1186/1756-0500-3-191

**Published:** 2010-07-13

**Authors:** Kelvin Li, Timothy B Stockwell

**Affiliations:** 1 The J. Craig Venter Institute, 9704 Medical Center Drive, Rockville, MD 20850, USA

## Abstract

**Background:**

High-throughput DNA sequencing has produced a large number of closed and well annotated genomes. As the focus from whole genome sequencing and assembly moves towards resequencing, variant data is becoming more accessible and large quantities of polymorphisms are being detected. An easy-to-use tool for quickly assessing the potential importance of these discovered variants becomes ever important.

**Findings:**

Written in Perl, the VariantClassifier receives a list of polymorphisms and genome annotation, and generates a hierarchically-structured classification for each variant. Depending on the available annotation, the VariantClassifier may assign each polymorphism to a large variety of feature types, such as intergenic or genic; upstream promoter region, intronic region, exonic region or downstream transcript region; 5' splice site or 3' splice site; 5' untranslated region (UTR), 3' UTR or coding sequence (CDS); impacted protein domain; substitution, insertion or deletion; synonymous or non-synonymous; conserved or unconserved; and frameshift or amino acid insertion or deletion (indel). If applicable, the truncated or altered protein sequence is also predicted. For organisms with annotation maintained at Ensembl, a software application for downloading the necessary annotation is also provided, although the classifier will function with properly formatted annotation provided through alternative means.

**Conclusions:**

We have utilized the VariantClassifier for several projects since its implementation to quickly assess hundreds of thousands of variations on several genomes and have received requests to make the tool publically available. The project website can be found at: http://www.jcvi.org/cms/research/projects/variantclassifier.

## Findings

The prevalence and increasing ubiquity of genome resequencing has greatly increased due to the emergence of lower cost deep sequencing technologies, such as Roche 454 [1] or Illumina Solexa [2], when compared to traditional Sanger sequencing. This has led to an enormous growth of variant data on well-characterized and annotated genomes. To manually filter through all the collected variant information is a daunting and error prone task, especially given the variety of information that may influence the assessment of a variant's importance. Winnowing through the possible information that can be derived from annotation is naturally a hierarchical process. For example, a single nucleotide polymorphism (SNP) in an intergenic region may be less interesting than one found in a gene, and a SNP found in an intron may be less interesting than one found in an exon. However, the intron/exon classification is irrelevant if the intergenic classification is already made.

Some variation annotation can be found at dbSNP [3] and Ensembl [4], after a variant has been submitted for publication, but no stand-alone or free tools currently exist for an investigator to quickly assess identified variants. We have developed VariantClassifier, an easy-to-use software tool that utilizes user-supplied genome annotation to classify variants hierarchically. When results have been loaded into a spreadsheet application, identified variants can be sorted by their classification, helping to prioritize the subsets of variants investigators may be interested in focusing on.

### Inputs

**Figure 1:**
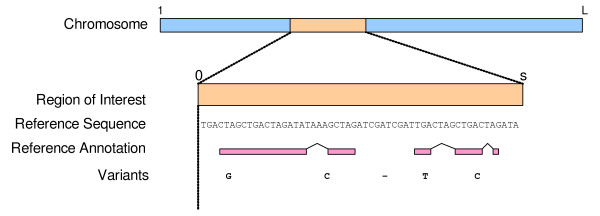
**Relationship between query variants, reference annotation and sequence in the region of interest, and the complete reference genome/chromosome**. For Ensembl genomes, the coordinate system is defined in 1-"residue"-based coordinates, from 1 to the length of the chromosome, *L*. The region of interest is a subset of the genome. The VariantClassifier uses a 0-"space"-based coordinate system to facilitate the description of indels. This local coordinate system ranges from 0 to the length of the reference sequence subregion, *s*. The reference annotation and query variants need to be specified in the region of interest's local coordinate system.

There are 3 inputs into the VariantClassifier. See Figure 1 for the relationship between the inputs and the organism's genome.

#### 1.) Reference nucleotide sequence FASTA file

The reference sequence provides the coordinate system for the reference annotation and the query variants in the region of interest. The sequence is also utilized to make a protein sequence prediction, if a variant occurs in the coding sequence of the transcript. For large genomes, the reference nucleotide sequence will be a subregion of the complete genome.

#### 2.) Reference annotation file

The reference annotation file, a tab-separated-value (TSV) text file, contains the positions of all the features annotated in the organism of interest in the local coordinates of the sequence file. For most investigators focused on a single organism or a single region of a genome, the annotation input will remain constant. For organisms, such as *Homo sapiens*, which has extensive annotation retrievable from Ensembl, a Perl application is included as part of the VariantClassifier package, which utilizes the Ensembl API to extract the annotation and nucleotide sequence file that the VariantClassifier application requires. For organisms without the benefit of the Ensembl resource, this annotation file will need to be generated using an alternative method, but only once.

#### 3.) Query variants file

The query variants file is a TSV text file consisting of each variant's position on the reference nucleotide sequence, orientation, and assayed allele. Since the coordinates of the query variants file are in local 0-space-based reference coordinates, the length of the allele on the reference is the distance between the specified begin and end coordinate. Therefore, if the mutant allele is the same length as the reference allele, a single nucleotide or block substitution is assumed. If the reference allele is shorter than the mutant allele, an insertion is assumed. A deletion is assumed if the reference allele is longer than the mutant allele. The information necessary to construct the query variants file is often available after reads have been mapped onto the reference genome. The format of this information will vary depending on the mapping software that has been chosen.

### Outputs

There are two text file outputs from the VariantClassifier. The information contained between the two outputs are the same, but they are formatted differently:

#### 1.) Normalized output

The normalized output should be read with an application that respects tab characters when displaying the text. The suggested viewer application could be a spreadsheet program such as OpenOffice Calc or Microsoft Excel. In this output format, information at the same classification hierarchy is displayed at the same tab stop position, or indentation distance.

#### 2.) Denormalized output

The denormalized output was designed to be utilized by line-based parsers. Each line in this format contains every level of classification that could be assigned to each variant.

### Implementation

**Figure 2:**
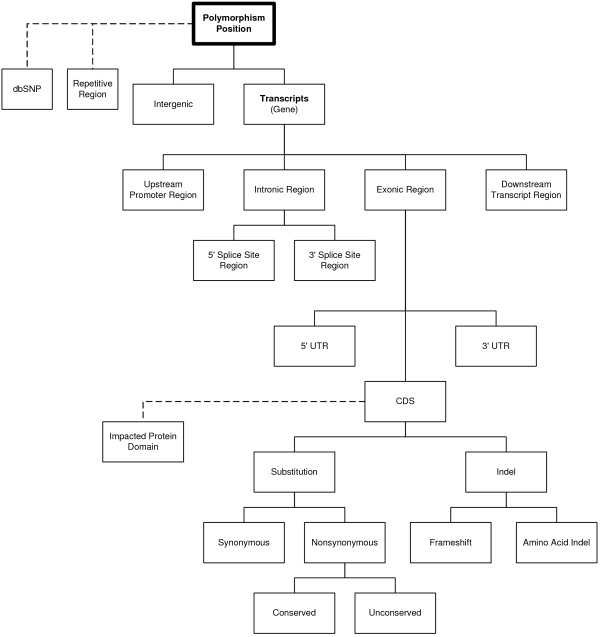
**VariantClassifier decision tree**. For each position/allele combination, the VariantClassifier uses this decision tree to make its hierarchical classification.

For every variant, a series of assessments are made according to the decision tree shown in Figure 2. The decision tree is traversed from the root, located at the top of the graph, towards a leaf node, where a branch terminates. The decision to traverse a node, and receive a classification, is based on the variant's positional overlap with a feature's position that was described in the reference annotation file.

For every variant, an initial assessment of the variant's novelty or overlap with a repeat is generated if it collocates with an existing dbSNP ID or an annotated repeat, respectively. If the variant does not overlap with a gene, then it is considered intergenic. For each alternative transcript associated with a gene, if the variant falls in a region 1,000 bases upstream of the first exon or downstream of the last exon, it is considered in an "upstream promoter region" or "downstream transcript region", respectively. A distance to the nearest exon associated with the promoter region is provided. Variants found between exons are considered "intronic", and a "5' Splice Site" or "3' Splice Site", is assigned if the variant is within 10 bases downstream or 6 bases upstream of an exon in its coding orientation. A distance to the nearest exon is provided. Variants considered "exonic" are further classified into "5' UTR", "CDS", or "3' UTR". Variants found in CDS have their positions mapped into amino acid coordinates, where they are assigned an "Impacted Protein Domain" based on Pfam annotation [5]. If the effect of the substitution is in a single codon, then the amino acid change is determined to be "synonymous" or "non-synonymous". Non-synonymous amino acid changes are classified as "Conserved" or "Unconserved." Indel variants are classified into "Frameshift" or "Amino Acid Indel", and a new protein sequence is predicted.

To determine whether variants causing non-synonymous substitutions are "conserved" or "unconserved", the BLOck SUbstitution Matrix (BLOSUM) [6] with a 30% cutoff is referenced. If the BLOSUM30 value for an amino acid transition is less than 0, then the substitution is considered "unconserved". Any amino acid shift to a stop codon is also considered "unconserved". Because this BLOSUM-based assessment is quick and convenient, it is also relatively naïve and we recommend using SIFT [7] for a more thorough follow up assessment.

Additionally, flanking sequence surrounding the variant is also provided for future assay design, or for the requisite context needed for dbSNP submission.

## Conclusions

To date, results from the VariantClassifier have contributed to the analyses of several large scale variant analyses publications [8,9]. The multi-level detailed nature of the output has made it possible to accurately assess the impact of novel variants quickly, effectively utilizing the annotation that is often available for organisms of interest that have been targeted for resequencing. The software is freely available on SourceForge.net.

## Availability and requirements

**Project name: **VariantClassifier

**Project home page: **http://www.jcvi.org/cms/research/projects/variantclassifier

**Sourceforge Download: **http://sourceforge.net/projects/variantclass

**Operating system: **Tested and in production on Linux.

**Programming language: **Perl

**License: **GNU GPL V3

**Any restrictions to use by non-academics: **none

## Competing interests

The authors declare that they have no competing interests.

## Authors' contributions

KL implemented the software. TBS and KL conceived of the study and validated the results. KL and TBS wrote the manuscript. All authors read and approved the final manuscript.
